# Crystal structure of rhodopsin in complex with a mini-G_o_ sheds light on the principles of G protein selectivity

**DOI:** 10.1126/sciadv.aat7052

**Published:** 2018-09-19

**Authors:** Ching-Ju Tsai, Filip Pamula, Rony Nehmé, Jonas Mühle, Tobias Weinert, Tilman Flock, Przemyslaw Nogly, Patricia C. Edwards, Byron Carpenter, Thomas Gruhl, Pikyee Ma, Xavier Deupi, Jörg Standfuss, Christopher G. Tate, Gebhard F. X. Schertler

**Affiliations:** 1 Laboratory of Biomolecular Research, Paul Scherrer Institute (PSI), 5232 Villigen PSI, Switzerland.; 2 Department of Biology, ETH Zürich, Wolfgang-Pauli-Strasse 27, 8093 Zürich, Switzerland.; 3 Medical Research Council Laboratory of Molecular Biology, Francis Crick Avenue, Cambridge CB2 0QH, UK.; 4 Fitzwilliam College, University of Cambridge, Cambridge, UK.; 5 Condensed Matter Theory Group, PSI, 5232 Villigen PSI, Switzerland.

## Abstract

Selective coupling of G protein (heterotrimeric guanine nucleotide–binding protein)–coupled receptors (GPCRs) to specific Gα-protein subtypes is critical to transform extracellular signals, carried by natural ligands and clinical drugs, into cellular responses. At the center of this transduction event lies the formation of a signaling complex between the receptor and G protein. We report the crystal structure of light-sensitive GPCR rhodopsin bound to an engineered mini-G_o_ protein. The conformation of the receptor is identical to all previous structures of active rhodopsin, including the complex with arrestin. Thus, rhodopsin seems to adopt predominantly one thermodynamically stable active conformation, effectively acting like a “structural switch,” allowing for maximum efficiency in the visual system. Furthermore, our analysis of the well-defined GPCR–G protein interface suggests that the precise position of the carboxyl-terminal “hook-like” element of the G protein (its four last residues) relative to the TM7/helix 8 (H8) joint of the receptor is a significant determinant in selective G protein activation.

## Teaser

The structure of the rhodopsin/mini-G_o_ complex reveals new insights on G protein selectivity.

## INTRODUCTION

G protein (heterotrimeric guanine nucleotide–binding protein)–coupled receptors (GPCRs) are a large family of membrane receptors that interact with heterotrimeric G proteins to transform extracellular signals into cellular responses. Selective coupling of GPCRs to specific Gα-protein subtypes is a critical step that determines their physiology and their response to natural ligands and clinical drugs. While previous studies have revealed regions on GPCRs and G proteins involved in selectivity (*1*, *2*), the molecular details remained elusive. These details can be elucidated by examining and comparing the structure of GPCR–G protein complexes, which lie at the center of this signal transduction event.

Crystal structures of GPCR signaling complexes have been determined only for the G_s_ protein and arrestin. Several techniques are being used to stabilize these GPCR–G protein complexes for structural determination, such as fusion of T4 lysozyme on the receptor and the use of a nanobody (*3*) or mini-G protein (*4*), or the creation of a fusion chimera in the rhodopsin-arrestin complex (*5*). Mini-G proteins comprise the guanosine triphosphatase (GTPase) domain of the α subunit of heterotrimeric G proteins and are fully capable of stabilizing GPCRs in their active state, generating characteristic changes in agonist affinity and recapitulating G protein specificity in vivo (*6*–*8*).

The light-sensitive visual receptor rhodopsin is the prototypical class A (rhodopsin-like) GPCR. It is activated by light-induced isomerization of the covalently bound ligand retinal. Upon activation, rhodopsin couples to and activates G_t_ (transducin), a member of the G_i/o_ subfamily (*2*), thus triggering the cyclic guanosine monophosphate cascade. At the center of this signal transduction mechanism lies the formation of a complex between active rhodopsin and the G protein. Rhodopsin is the first GPCR that had structures determined in the inactive and active states with and without agonist (*9*) and in an arrestin-bound state (*5*). In addition, rhodopsin is the only GPCR for which structures of human disease mutants have been solved (*10*, *11*). Comparisons of these structures revealed that rhodopsin activation results in the opening of a cleft on the cytoplasmic side of the receptor and the formation of a binding cavity for the C-terminal domain (α5) of G_t_. The rhodopsin system has significantly contributed to our understanding of class A GPCR activation (*12*–*16*).

The organization of rhodopsin and the G proteins G_i_ and G_t_ in the signaling complex has been recently analyzed by combining site-directed spin labeling and electron paramagnetic resonance spectroscopy data with molecular dynamics simulations (*17*, *18*). In addition, a mutagenesis analysis performed with native rhodopsin and G_i_ allowed for the location of residues involved in the stabilization of the complex (*19*). While these results have provided valuable information on the overall arrangement of the complex, a high-resolution, three-dimensional (3D) map of the interaction interface is still missing. Obtaining additional structures with this G protein subtype will allow us to identify the structural elements defining G protein selectivity.

## RESULTS

**Fig. 1:**
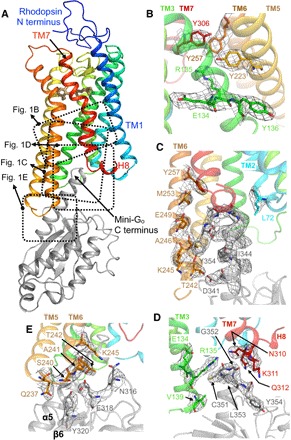
Structure of the rhodopsin/mini-G_o_ complex. Rhodopsin is colored in a rainbow spectrum with the N terminus in blue and the C terminus in red. Mini-G_o_ is shown in gray. Side chains are shown as sticks with carbon atoms in the same color as the backbone, oxygen in red, nitrogen in blue, sulfur in yellow, and the agonist all-trans retinal in light brown. Electron densities of the 2*F*_obs_-*F*_calc_ map are contoured in a gray mesh at a 1.5σ cutoff. (**A**) Overall view of the rhodopsin/mini-G_o_ complex. Specific regions are detailed in the adjacent panels. (**B**) The ionic interaction between E134^3.49^ and R135^3.50^ at the D/ERY motif is broken; opening of this interhelical ionic lock is one of the hallmarks of GPCR activation. The constitutively activating M257^6.40^Y mutation assists in stabilizing the extended active conformation of R135^3.50^, as observed in the structure of this mutant bound to a G_t_-derived peptide (*20*). (**C**) Contact interface between the C terminus of mini-G_o_ and TM2 and TM6 in the receptor. (**D**) Contact interface between the C terminus of mini-G_o_ and TM3 and TM7 in the receptor. The last four residues of the G protein (C-terminal hook) are located between R135^3.50^ and the joint between TM7 and helix 8 (H8). (**E**) Contact interface between the β6 sheet in mini-G_o_ and intracellular loop 3 (ICL3) in the receptor.

Here, we report the crystal structure of the stabilized constitutively active mutant of bovine rhodopsin N2C/M257Y/D282C in complex with mini-G_o_. These three mutations do not affect negatively the structure or functional properties of active rhodopsin (*20*). Attempts at creating a mini-G protein from G_t_ were unsuccessful, and therefore, we used the related G_o_ (62% sequence identity) (*6*). Similar to the full heterotrimeric G protein (*21*), mini-G_o_ couples effectively to light-activated rhodopsin solubilized in detergent, creating a stable complex that shows the characteristic spectral properties of the fully active state of rhodopsin bound to all-trans retinal (fig. S1A). The complex was purified in octyl glucose neopentyl glycol (OGNG) and crystallized by vapor diffusion at 4°C. Merging diffraction data from five crystals allowed structure determination by molecular replacement and refinement up to a resolution of 3.1 Å (Material and Methods, table S1, and fig. S2). Crystals grew with *p*6_1_ symmetry, in which the crystal packing is mainly mediated by hydrophilic contacts between mini-G_o_ molecules. The orthosteric ligand-binding pocket contains only partial density for the agonist all-trans retinal (fig. S3), but the electron density is well defined at the mini-G_o_ binding interface in the cytoplasmic cleft (Fig. 1).

### Mini-G_o_ binding interface to rhodopsin

**Fig. 2:**
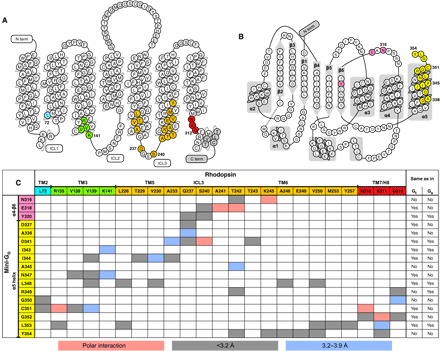
Contact interface between rhodopsin and mini-G_o_. (**A**) Secondary structure of bovine rhodopsin. Residues that are within 3.9 Å of mini-G_o_ are highlighted in colors matching those in Figs. 1A, 2C, and 4A. The gray regions are not determined in our structure. (**B**) Secondary structure of mini-G_o_. Residues that are within 3.9 Å of rhodopsin are highlighted in colors matching those in Figs. 2C and 4A. The dashed line represents the region that is not determined in the structure. (**C**) Table displaying the residue-residue contacts and distances between rhodopsin and mini-G_o_; the right two columns show for each residue in mini-G_o_ if the corresponding position in G_t_α1 (UniProtKB P04695) and G_s_α (UniProtKB P04896) holds the same residue or not. Residue-residue interactions are colored blue (3.2 to 3.9 Å) and gray (shorter than 3.2 Å), while polar interactions are labeled in salmon pink. The rhodopsin and mini-G_o_ models used to calculate the distances contained virtual hydrogen atoms added during structure refinement.

The crystal structure of the engineered G protein mini-G_o_ bound to rhodopsin displays a high overall similarity to mini-G_s_ bound to the adenosine A_2A_ receptor [A_2A_R; root mean square deviation (RMSD), 1.11 Å] (*4*), as well as to the guanosine triphosphate (GTP)–binding domains of heterotrimeric G_s_ bound to the β_2_-adrenergic receptor (β_2_AR; RMSD, 1.17 Å) (fig. S4A) (*3*). The protein-protein interfaces of mini-G_o_ and rhodopsin in the complex are composed of 17 residues and 23 amino acid residues (Fig. 2), with a surface area of 1104 and 1375 Å^2^, respectively. An evolutionary analysis (*22*) classifies this interface as biological (that is, not as merely a crystallographic contact). The regions on mini-G_o_ that make contact with the receptor comprise the α4-β6 loop, the β6 sheet, and the α5 helix (Figs. 1 and 2 and fig. S5). In the receptor, the major contact regions to mini-G_o_ are located across the cytoplasmic ends of transmembrane helices TM2, TM3, TM5, TM6, and the TM7/H8 joint, with additional contacts mediated by the ICL3. These contact interfaces agree with a previous cross-linking study using rhodopsin mutants and native transducin (*23*) and with a mutagenesis analysis performed with native rhodopsin and G_i_ mutants (*19*). These data, combined with the structural similarities to existing GPCR–G protein complexes, strongly suggest that the complex between rhodopsin and mini-G_o_ recapitulates the main features (orientation and contact interface) of the native interaction with Gα_t_.

**Fig. 3:**
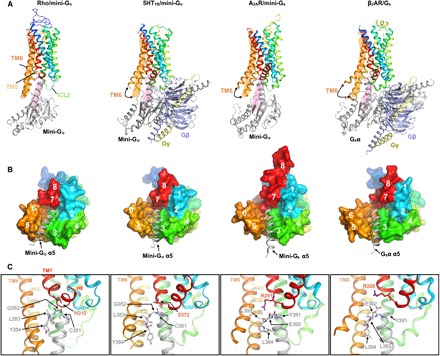
Comparison of the G protein binding interface in GPCR–G protein complexes. Structures of rhodopsin/mini-G_o_, 5HT_1B_R/mini-G_o_ (PDB ID: 6G79), A_2A_R/mini-G_s_ (PDB ID: 5G53), and β_2_AR/G_s_ (PDB ID: 3SN6) superimposed to the Cα atoms of rhodopsin. Receptors are colored in rainbow spectrum. (**A**) Side view of the complex structures. The orange and pink areas serve as a reference for the locations of TM6 in rhodopsin and the C-terminal α5 helix of mini-G_o_ bound to rhodopsin, respectively. (**B**) Cytoplasmic view of the binding interface between receptors and G proteins. The receptors are shown as surfaces, and the C-terminal α5 helices of G proteins are shown as gray helices. The numbers mark the positions of the cytoplasmic end of TM1 to TM7 and H8. (**C**) Detailed view of the contact interface between the TM7/H8 joint (N310^8.47^ in rhodopsin, S372^7.56^ in 5HT_1B_R, R291^7.56^ in A_2A_R, and R328^7.55^ in β_2_AR) and the C-terminal hook (CGLY in G_o_ and YELL in G_s_).

**Fig. 4:**
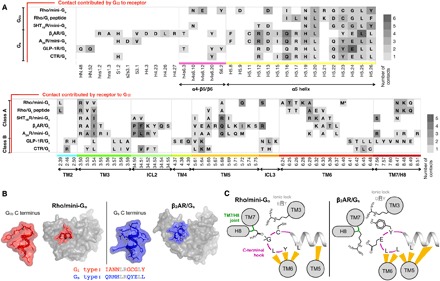
Comparison between contact interfaces in GPCR–G protein complexes. (**A**) Contact regions between receptors and Gα. We compare seven complex structures: rhodopsin/mini-G_o_, rhodopsin/G_t_ peptide (PDB ID: 4A4M), 5HT_1B_R/mini-G_o_ (PDB ID: 6G79), β_2_AR/G_s_ (PDB ID: 3SN6), A_2A_R/mini-G_s_ (PDB ID: 5G53), glucagon-like peptide 1 receptor (GLP-1R)/G_s_ (PDB ID: 5VAI), and calcitonin receptor/G_s_ (PDB ID: 5UZ7). We define contacts if two residues are closer than 4 Å. We color each residue according to the number of residue contacts established with the interacting partner. In the lower panel, M* depicts the stabilizing mutation M257Y in rhodopsin. (**B**) 3D shape of the last 12 residues of Gα (mini-G_o_ in red and G_s_ in blue) when bound to active rhodopsin and β_2_AR (gray surfaces). The sequences of the C-terminal elements are shown below, with conserved residues colored gray. (**C**) The C-terminal hook is a determinant of GPCR–G protein selectivity. The yellow strokes depict contacts within 4 Å between G protein and receptor.

The C-terminal portion of the α5 helix in mini-G_o_ contributes most of the interactions to the receptor, as previously observed in G_s_ coupled to other GPCRs (Figs. 2C, 3, and 4A). In contrast, the contacts that we observe between the β6 sheet and the receptor are not present between either A_2A_R (*4*) or β_2_AR (*3*) and G_s_; this is probably a consequence of the different modes of binding of G_o_ to rhodopsin (see below) (*18*). In addition, the contacts between the helical ICL2 of A_2A_R, β_2_AR, and the serotonin 5-HT_1B_ receptor (5-HT_1B_R) (*24*) and the β2-β3 loop/β3 sheet in the α subunit of G_s_ are absent in the rhodopsin/mini-G_o_ structure, in which ICL2 is unstructured (Fig. 3A). Thus, these differences may reflect a certain degree of plasticity in the interface between GPCRs and G proteins outside the α5 helix–binding crevice.

The residues in rhodopsin involved in making contacts with mini-G_o_ are also very similar to those between A_2A_R or β_2_AR and G_s_, although there are some subtle differences (Fig. 4A). Only in rhodopsin and class B GPCRs are there contacts mediated by TM2. Moreover, the contacts with TM7/H8 are more prominent in G_o_- than in G_s_-coupled complexes. There are also differences in the number of residues in each structural element making contact to the α subunit. In particular, the smaller outward movement of TM6 in the rhodopsin/mini-G_o_ structure, coupled with the different tilt of the α5 helix in the G protein, results in most of the rhodopsin/mini-G_o_ contacts being in TM6 and fewer in TM5 (Figs. 1C, 3, and 4A). In contrast, in the β_2_AR/G_s_ structure and the A_2A_R/mini-G_s_ structure, most of the contacts are observed in TM5 and the end of TM3 running into ICL2 (Figs. 3 and 4A).

In summary, the contact residues in the complex interfaces spread over the cytoplasmic region of the receptors, and we cannot observe concrete interaction patterns. Thus, the selectivity between receptors and G proteins is not just encoded in the 2D protein sequence of the effector-binding site but rather in its 3D shape.

### Selectivity between receptor and G protein

The major difference between the G_o_- and G_s_-GPCR complexes lies in the pose of the C-terminal α5 domain within the effector-binding site, with a difference in tilt angle of about 30° (Fig. 3 and fig. S4B). Remarkably, the last four amino acid residues of the α5 helix in G_i/o_ and G_s_ fold in an identical “hook-like” structure upon binding the receptor (*3*, *4*, *20*). However, their different side chains mold these elements into dissimilar shapes (Fig. 4B). This short C-terminal hook and the adjacent residues in α5 all make contacts to the receptor and are the most important structural elements in defining the subtype specificity of G proteins (*2*, *25*–*27*).

The degree of opening in the cytoplasmic binding cavity partially determines the overall placement of the C-terminal hook, as its last two residues interact with the “displaced” cytoplasmic domains of TM5/6 (Figs. 3C and 4C). This hook also latches to TM3 [a structural hub of the GPCR fold located in the opposite side of the binding cavity (*28*)] by a subtype-specific interaction [G_i/o_, Cys351^H5.23^; G_s_, Tyr391^H5.23^; the residue numbering refers to the human wild-type G_o1_α subunit (UniProtKB P09471), and the superscripts refer to the common Gα numbering system (*25*)] with Arg^3.50^ [conserved in class A GPCRs; superscripts in receptor residue numbers refer to the Ballesteros-Weinstein general numbering scheme (*29*)]. As a result, the hook locates at the interface between TM5/6 and the TM7/H8 joint in the binding cavity of the receptor. We observed that the conserved Gly352^H5.24^ of mini-G_o_ forms a backbone–side chain interaction with Asn310^8.47^ in the receptor, placing it very close to the TM7/H8 joint (Fig. 3C). In contrast, G_s_ has a much bulkier Glu392^H5.24^, which rests between the TM7/H8 turn and the TM5/6, interacting with Lys270^6.32^ (Figs. 3C and 4C). As a result, the relative orientation between the C-terminal hook and TM7/H8 of the receptor is the main factor that differentiates the binding of G_i/o_ and G_s_. Thus, GPCRs could modulate their selectivity for G proteins by shaping the cytoplasmic binding cavity to fit the C-terminal hook in a specific pose that results in a pulling motion of the α5 helix in the G protein, leading to its activation (*30*, *31*). In G_i_, this pose requires that the C-terminal hook packs its backbone tightly to the TM7/H8 turn. In contrast, recognition of the G_s_ C-terminal hook involves a structurally looser interaction with the TM7/H8 joint and TM5/6.

### Two conformationally distinct states of rhodopsin

The reaction pathway of rhodopsin activation has been delineated in great detail using a variety of spectroscopy methods, revealing the existence of several identifiable intermediate states (*15*, *16*). Furthermore, the structure of some of these intermediates has been determined by x-ray and electron crystallography (*9*). The early agonist-bound intermediates batho, lumi, and meta I form in microseconds and show only minor structural changes limited to the ligand-binding pocket. Similar small and local changes have also been observed in other agonist-bound inactive-state GPCR structures (*32*, *33*). In contrast, transition to later stages in rhodopsin activation (meta IIa, meta IIb, and meta IIbH^+^) involves the opening of a cytoplasmic effector-binding site within a few milliseconds through the relocation of the cytoplasmic end of TM6. The equilibrium between these late active states is partially regulated by subtle changes, such as in the protonation state at the cytoplasmic D/ERY motif (ionic lock) (*13*) or in the electronic properties of retinal (*34*). All identified spectroscopic states of rhodopsin fall into two main structural categories: inactive (including the inverse agonist-bound state and the agonist-bound early active intermediates) and active (apoprotein at low pH, agonist-bound late active states and arrestin-bound rhodopsin). The active conformation of rhodopsin displays a relatively small opening in the cytoplasmic effector-binding site. To date, it has been unclear whether this was a true feature of rhodopsin bound to G_t_ and, by extension, of other GPCRs bound to G_i/o_.

**Fig. 5:**
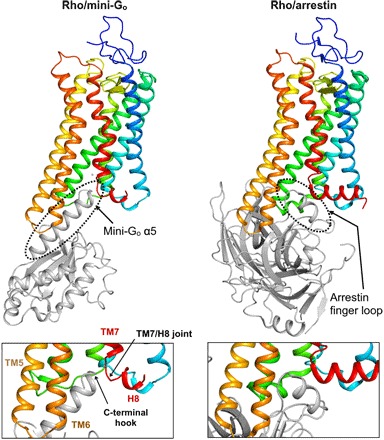
Structural comparison between rhodopsin complexes. (**Top**) Comparison of the rhodopsin/mini-G_o_ complex (left) and the arrestin-bound structure (PDB ID: 5W0P; right) shows that rhodopsin exposes an identical site for the recognition of the α5 helix of Gα and the finger loop of arrestin. (**Bottom**) Detail of the G protein and arrestin binding interfaces viewed parallel to the cell membrane.

**Fig. 6:**
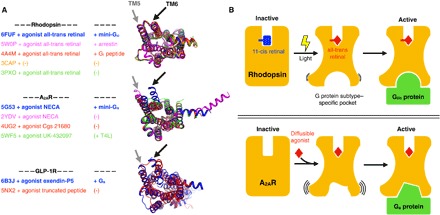
Conformational changes upon GPCR activation. (**A**) Comparison between active conformations of rhodopsin, A_2A_R, and GLP-1R, respectively. We chose these receptors because their structures have been solved in different active and intermediate-active states. The superimposed structures are shown from the cytoplasmic side. We highlight the location of TM5 and TM6 to assist in visualizing the opening of the effector-binding site. (**B**) The top panel depicts the structural intermediates during rhodopsin activation. Light-induced retinal isomerization results in the formation of a predefined binding pocket for a G_i/o_ protein or arrestin. On the other hand, the bottom panel depicts how binding of diffusible ligands results in the formation of transient active GPCR conformations with higher conformational heterogeneity than active rhodopsin. In this case, the G protein likely selects and stabilizes a suitable conformation of the binding pocket from this ensemble.

Surprisingly, the structure of rhodopsin in complex with mini-G_o_ is virtually identical to all the previous structures of active rhodopsin (average RMSD, 0.51 ± 0.07 Å; Figs. 5 and 6A and table S2) (*5*, *20*, *35*–*38*). Thus, our results show that a GPCR can expose an identical site for the recognition of the α5 helix of Gα and the finger loop of arrestin (Fig. 5). Rhodopsin thus effectively acts like a structural switch upon activation, adopting predominantly two thermodynamically stable conformational states, an inactive state and an active state. In contrast, spectroscopic and structural studies suggest that GPCRs that bind diffusible ligands exist as an ensemble of conformations that include a number of inactive, intermediate, and active conformations (*1*, *39*). Accordingly, their structures show a higher degree of variability (Fig. 6A).

## DISCUSSION

The crystal structure of rhodopsin bound to an engineered mini-G_o_ protein provides new insights into the molecular basis of GPCR–G protein recognition and selectivity. Comparison with existing complexes suggests that the sequence of the binding interface alone does not determine selectivity (Fig. 4A). On the other hand, we notice that the interactions between ICL2 and ICL3 of the GPCRs and the GTPase domain of G proteins show a certain degree of plasticity, which could account for selective protein-protein interactions. In addition, the different extent in the movement of TM6 of GPCRs upon activation has been proposed to play a fundamental role in G protein selectivity (*30*, *31*). While all these factors probably contribute to pulling the α5 helix to selectively activate specific G proteins, we suggest that another significant determinant in this mechanism is the precise placement of the C-terminal hook-like element of the G protein (its last four residues) relative to the TM7/H8 joint.

Activation of GPCRs involves a precise coupling between the physicochemical properties of their ligands and the dynamic properties of the receptor that results in the engagement and activation of intracellular signaling proteins to promote downstream signaling. GPCRs that bind diffusible ligands have been shown to exist in an ensemble of conformations (Fig. 6B) (*40*). We show here that the visual receptor rhodopsin, on the other hand, functions like a “conformational switch” upon activation. This behavior remains even when the covalently bound ligand retinal is replaced by diffusible, pharmacologically designed compounds (fig. S6) (*41*), suggesting that this is an inherent property of the receptor. This remarkable property of rhodopsin is likely related to the control of visual perception. First, a stable inactive conformation (in combination with other adaptations such as the presence of high local concentrations of both rhodopsin and G proteins in the rod photoreceptor membranes) allows for a tight control of basal activity, which is critical to reduce dark noise in visual systems. In addition, by having a unique active conformation, binding to G proteins and the turnover time for allowing arrestin binding after receptor phosphorylation are probably optimized, allowing for the activation of about 600 G_t_ molecules per second (*42*).

The structure of rhodopsin bound to mini-G_o_ presented here adds a key piece to the gallery of states along its activation pathway, making rhodopsin the best-described GPCR in terms of structure. We can now sequentially track the conformations of a GPCR from its inactive state to agonist-induced activation, G protein coupling, and binding to arrestin. Furthermore, the structure provides new insights into the binding interface between activated GPCRs and G_i/o_ proteins. This information advances our understanding of GPCR–G protein selectivity and how GPCR conformations promote certain signaling pathways.

## MATERIALS AND METHODS

### Protein preparation

Mini-G_o_ was prepared as described (*6*, *43*). The N2C/M257Y/D282C mutant of bovine rhodopsin was expressed in human embryonic kidney (HEK) 293 GnTI^−^ cells as described (*20*, *44*). Buffers for every purification step were cooled to 4°C before use, and the steps after the addition of 9-cis retinal were performed under dim red light before light activation of rhodopsin. HEK293 cells were homogenized in phosphate-buffered saline (PBS) buffer and then solubilized by supplementing dodecyl maltoside (DDM) (Sol-Grade, Anatrace) to 1.25% (w/v). After gentle mixing for 1 hour, the cell lysate was centrifuged at 200,000*g* for 1 hour to remove unsolubilized residual. The rhodopsin apoprotein in the solubilisate was captured using immunoaffinity agarose resin coupled to 1D4 antibody. The resin was collected and washed with 10 column volume (CV) of PBS buffer containing 0.04% DDM, and then 2 CV of PBS buffer containing 0.04% DDM and 50 mM 9-cis retinal (Sigma-Aldrich) or 11-cis retinal (National Institutes of Health and Hoffmann–La Roche) was mixed with the resin overnight in the dark. The resin was collected and washed sequentially with 15 CV of PBS buffer containing 0.04% DDM; 10 CV of buffer containing 20 mM Hepes (pH 7.5), 100 mM NaCl, and 0.12% (w/v) OGNG (Anatrace); and 5 CV of buffer containing 20 mM Hepes (pH 7.5), 50 mM NaCl, 1 mM MgCl_2_, and 0.12% OGNG. Dark-state rhodopsin was eluted thrice with 1.5 CV of buffer containing 20 mM Hepes (pH 7.5), 50 mM NaCl, 1 mM MgCl_2_, 0.12% OGNG, and 80 μM 1D4 peptide TETSQVAPA for more than 2 hours each time. Eluted protein was concentrated using an Amicon Ultra concentrator [molecular weight cutoff (MWCO), 50,000] to 5 to 10 mg/ml. The concentrated protein was subjected to deglycosylation using the endoglycosidase F1 (Endo F1) at 1:100 (w/w) ratio overnight at 4°C. The deglycosydated rhodopsin was mixed with mini-G_o_ at the molar ratio of 1:1.2 in the presence of apyrase (25 mU/ml; New England BioLabs), incubated for 30 min, and then irradiated with light passed through a 495-nm long-pass filter, followed by another 30-min incubation in the dark. The rhodopsin/mini-G_o_ mixture was concentrated using an Amicon Ultra concentrator (MWCO, 50,000) and subjected to size exclusion chromatography on a Superdex 200 Increase 10/300 GL column (GE Healthcare) using a buffer containing 20 mM Hepes, 0.4 mM MgCl_2_, and 0.12% OGNG. The eluted fractions were examined by SDS–polyacrylamide gel electrophoresis (PAGE) (fig. S1D), and those fractions containing pure rhodopsin/mini-G_o_ complex were pooled and concentrated to 8 mg/ml for crystallization. The ultaviolet-visible spectrum of the final sample was measured to optically evaluate the isoform of retinal in rhodopsin, showing an OD_280_/OD_380_ (ratio of optical density between 280 and 380 nm) of 1.8 in rhodopsin/mini-G_o_ in contrast to 1.6 in light-activated rhodopsin.

The detergent OGNG was selected from a screening experiment, where rhodopsin was stable in both the dark and light states and upon mini-G_o_ binding. Deglycosylation was a critical step for obtaining crystals of the rhodopsin/mini-G_o_ complex. Peptide *N*-glycosidase F and Endo F1 were tested, and only Endo F1 rendered a homogeneous deglycosylated product, which was confirmed by SDS-PAGE (fig. S1C).

### Crystallization and synchrotron data collection

Crystallization of the gel-filtrated rhodopsin/mini-G_o_ complex was set up using the vapor diffusion method. Crystallization drops were dispensed using a mosquito crystallization robot by mixing 200 nl of protein and 200 nl of crystallization buffer containing 0.1 M MES (pH 5.5) and 10 to 20% polyethylene glycol 4000 (PEG 4000) in an MRC 2-well crystallization plate (Swissci) at 4°C. Sword- or rod-shaped crystals appeared in 1 to 3 days, and crystals wider than 10 μm mainly grew from drops using the crystallization buffer containing 13 to 17% PEG 4000. Before crystals were harvested on crystal loops (MiTeGen), crystals were cryoprotected by adding 400 nl of mother liquor supplemented with 10% glycerol or 10% trehalose to the crystallization drop 1 day before harvesting. Harvested crystals were flash-frozen in liquid nitrogen (fig. S1B). Frozen crystals were evaluated at the PXI beamline at the Swiss Light Source (SLS) using raster scanning to identify the best diffracting locations on a frozen crystal. Diffraction datasets were collected by exposing crystals to a 10- or 20-μm-sized beam with 0.05° oscillation angle per frame.

### Refinement and model building

Individual diffraction datasets were analyzed using XDS (*45*) for integrating Bragg peaks. All datasets showed the same unit cell dimensions with negligible deviation (less than 0.5%) and space group *p*6_1_. The best five datasets were chosen for merging together to improve the signal-to-noise ratio. The datasets were combined using XSCALE without scaling and merging, and the pooled reflection list was further analyzed using the STARANISO server (Global Phasing Ltd.). The STARANISO server first analyzed the anisotropy for each dataset, giving a resolution of 3.69 Å in the *hk* plane and 3.11 Å in the *l* direction using the criterion of CC^1^/_2_ = 0.3. The scaling factor was determined and applied after applying the anisotropic mask. After scaling, the datasets were merged, and the final resolution was improved to 3.02 Å and anisotropically truncated to 3.12 Å in the best direction (table S1). Structures of rhodopsin (PDB ID: 4A4M) and mini-G_s_ (PDB ID: 5G53) were used as search models to perform molecular replacement with the program Phaser. The sequence of mini-G_o_ was modeled using the Swiss-Prot server (*46*). Structure refinement was performed using phenix.refine (*47*) and Rosetta refinement (*48*) from the Phenix suite. Manual model-building and adjustment of the coordinates were performed using the visualization program Coot. Polder maps (*49*) to verify the presence of retinal were calculated with Phenix using a low-resolution cutoff of 20 Å. For the retinal-only polder omit map, three cross-correlations were calculated between the following maps: (i) calculated *F*_obs_ with and without ligand (0.70), (ii) calculated and real *F*_obs_ with ligand (0.85), and (iii) calculated and real *F*_obs_ without ligand (0.77). These results verified the presence of retinal (fig. S3). In the *F*_obs_-*F*_calc_ (observed and calculated structure factors) map, there is a strong peak density above the 5σ cutoff, and a water molecule was accordingly modeled into this position. The outlier in the Ramachandran plot (R206 in the β3-α2 loop of mini-G_o_) was likely caused by a crystal contact with ICL3 of rhodopsin in a symmetry-related complex molecule.

### Comparison and analysis of structures

Structural models were downloaded from the Protein Data Bank. RMSDs (table S2) were obtained by superimposing the 3D structures with the super command in PyMOL (Schrödinger). The contacts between receptors and G proteins (Figs. 2 and 4A) were identified by selecting atoms within <4 Å cutoff. Figures were prepared using PyMOL.

## Supplementary Material

http://advances.sciencemag.org/cgi/content/full/4/9/eaat7052/DC1

## SUPPLEMENTARY MATERIALS

Supplementary material for this article is available at http://advances.sciencemag.org/cgi/content/full/4/9/eaat7052/DC1 Fig. S1. Sample preparation and crystals of the rhodopsin/mini-G_o_ complex. Fig. S2. Data completeness versus resolution. Fig. S3. Electron density of the all-trans retinal. Fig. S4. Structural comparison of the G proteins. Fig. S5. Sequence alignment of G_i/o_ proteins, mini-G_o_, and G_t_ peptide. Fig. S6. Comparison of active rhodopsin structures. Table S1. Statistics of the crystallographic data and the structural refinement. Table S2. Quantitative measure of similarity between active rhodopsin structures.
