# Supplementary material for: Crystal structure of rhodopsin in complex with a mini-Go sheds light on the principles of G protein selectivity
Source: Sci Adv. 2018 Sep 19;4(9):eaat7052. doi: 10.1126/sciadv.aat7052 (PMC6154990; doi:10.1126/sciadv.aat7052)
Supplement: http://advances.sciencemag.org/cgi/content/full/4/9/eaat7052/DC1 [file supp_4_9_eaat7052__index.html]

Science Advances | Science Advances

## Supplementary Materials

**This PDF file includes:**

- Fig. S1. Sample preparation and crystals of the rhodopsin/mini-Go complex.
- Fig. S2. Data completeness versus resolution.
- Fig. S3. Electron density of the all-trans retinal.
- Fig. S4. Structural comparison of the G proteins.
- Fig. S5. Sequence alignment of Gi/o proteins, mini-Go, and Gt peptide.
- Fig. S6. Comparison of active rhodopsin structures.
- Table S1. Statistics of the crystallographic data and the structural refinement.
- Table S2. Quantitative measure of similarity between active rhodopsin structures.

Download PDF

**Files in this Data Supplement:**

- Adobe PDF - aat7052\_SM.pdf
