# Supplementary material for: Crystal structure of rhodopsin in complex with a mini-Go sheds light on the principles of G protein selectivity
Source: Sci Adv. 2018 Sep 19;4(9):eaat7052. doi: 10.1126/sciadv.aat7052 (PMC6154990; doi:10.1126/sciadv.aat7052)
Supplement: http://advances.sciencemag.org/cgi/content/full/4/9/eaat7052/DC1 [file aat7052_SM.pdf]

Science Advances
AAAS

advances.sciencemag.org/cgi/content/full/4/9/eaat7052/DC1

# Supplementary Materials for

## Crystal structure of rhodopsin in complex with a mini-$G_o$ sheds light on the principles of G protein selectivity

Ching-Ju Tsai*, Filip Pamula, Rony Nehmé, Jonas Mühle, Tobias Weinert, Tilman Flock, Przemyslaw Nogly, Patricia C. Edwards, Byron Carpenter, Thomas Gruhl, Pikyee Ma, Xavier Deupi, Jörg Standfuss, Christopher G. Tate, Gebhard F. X. Schertler*

*Corresponding author. Email: ching-ju.tsai@psi.ch (C.-J.T.); gebhard.schertler@psi.ch (G.F.X.S.)

Published 19 September 2018, *Sci. Adv.* **4**, eaat7052 (2018)
DOI: 10.1126/sciadv.aat7052

**This PDF file includes:**

Fig. S1. Sample preparation and crystals of the rhodopsin/mini-$G_o$ complex.
Fig. S2. Data completeness versus resolution.
Fig. S3. Electron density of the all-trans retinal.
Fig. S4. Structural comparison of the G proteins.
Fig. S5. Sequence alignment of $G_{i/o}$ proteins, mini-$G_o$, and $G_t$ peptide.
Fig. S6. Comparison of active rhodopsin structures.
Table S1. Statistics of the crystallographic data and the structural refinement.
Table S2. Quantitative measure of similarity between active rhodopsin structures.

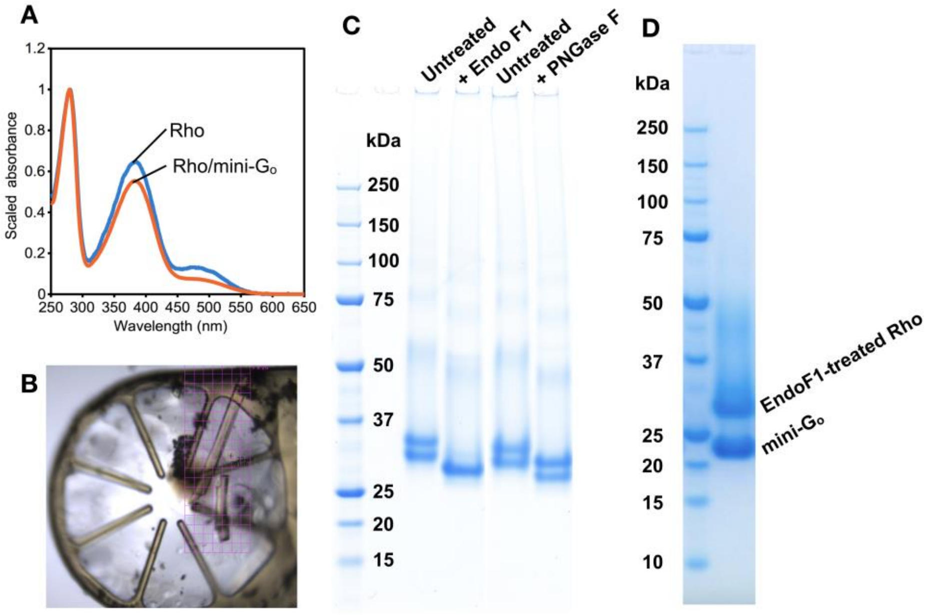


**Fig. S1. Sample preparation and crystals of the rhodopsin/mini-$G_o$ complex.** (**A**) UV-VIS spectra of rhodopsin (Rho) purified from the 1D4 immunoaffinity column and of rhodopsin/mini-$G_o$ complex purified from the Superdex 200 Increase 10/300 GL column. The values of $OD_{280}/OD_{380}$ are 1.6 for light-activated rhodopsin and 1.8 for the rhodopsin/mini-$G_o$ complex. (**B**) Flash-frozen crystals were harvested on a MiTeGen MicroGripper loop for exposure to synchrotron radiation. The pink mesh depicts the area used in the raster scanning for finding a well-diffracting location. Each pink square is 20 μm x 20 μm. (**C**) SDS-PAGE of the deglycosylation products of rhodopsin purified from the 1D4-immunoaffinity chromatography using Endo F1 and PNGase F. (**D**) SDS-PAGE of the rhodopsin/mini-$G_o$ complex purified from the Superdex 200 Increase 10/300 GL column.

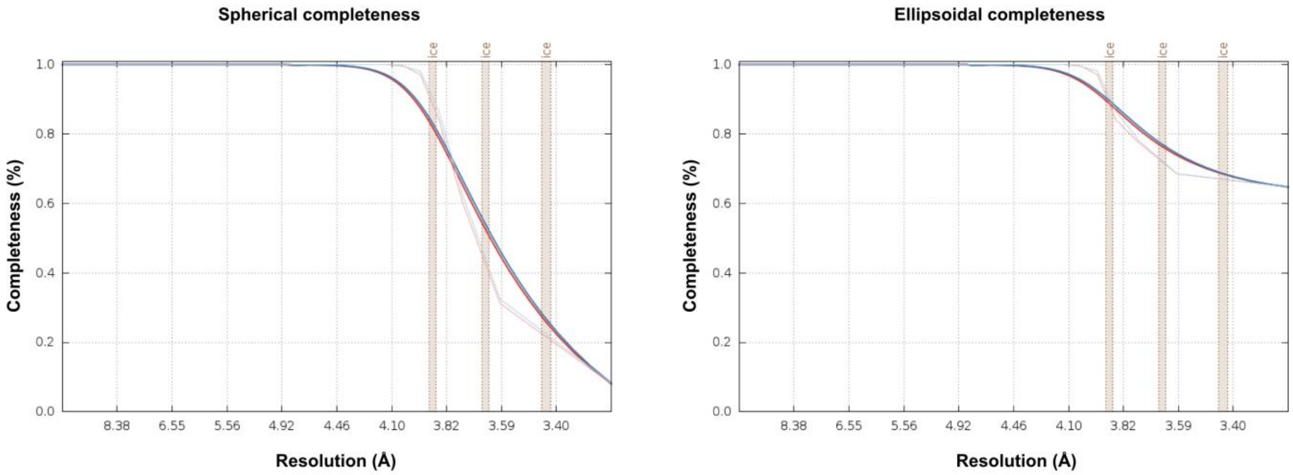


**Fig. S2. Data completeness versus resolution.** Spherical data completeness is displayed on the left and ellipsoidal completeness after data truncation is displayed on the right. The blue and red lines indicate anomalous and overall completeness, respectively. Thick lines are smooth approximations of the raw data (thin lines).

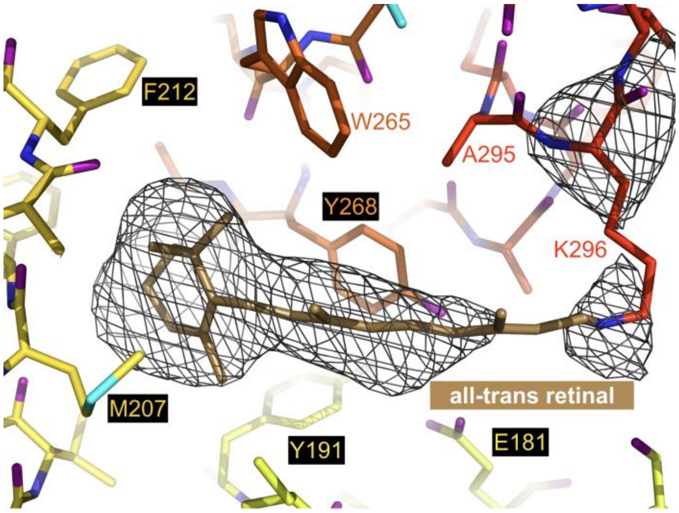


**Fig. S3. Electron density of the all-trans retinal.** The polder omit map of the agonist all-trans retinal and K296 of rhodopsin is displayed at 3.0 σ cutoff. The map density is sufficient to define the position of the β-ionone ring and roughly model the all-trans retinal in the structure. The colors of carbon atoms in each amino acid correspond to the positions of the amino acids as in Fig. 1A. The sticks represent oxygen in magenta, nitrogen in dark blue, sulfur in cyan, and all-trans retinal in light brown.

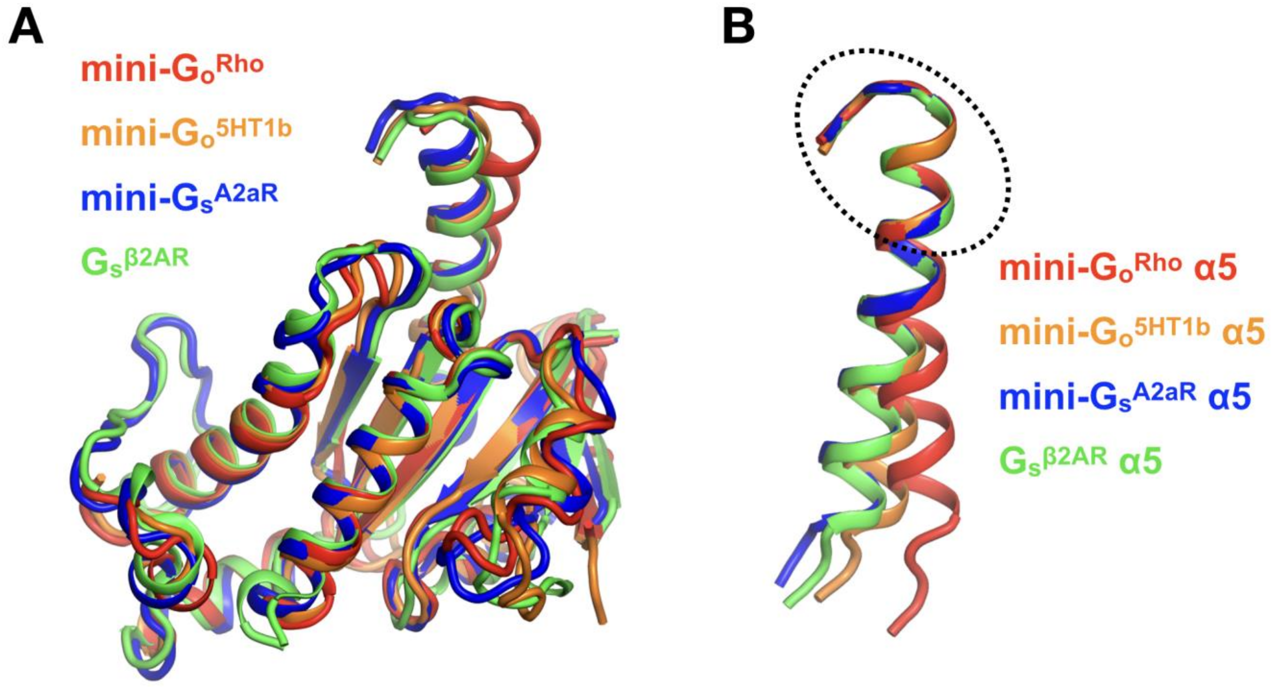


**Fig. S4. Structural comparison of the G proteins.** (**A**) Superposition of rhodopsin-bound mini-$G_o$ (red), $5HT_{1B}$R-bound mini-$G_o$ (PDB ID: 6G79, orange), $A_{2A}$R-bound mini-$G_s$ (PDB ID: 5G53, blue) and the $G_s\alpha$ nucleotide binding domain bound to $\beta_2$AR (PDB ID: 3SN6, green). The molecules are aligned to the Cα atoms of rhodopsin-bound mini-$G_o$. (**B**) Superposition of the C-terminal α5 helix of the G proteins listed in fig. S4A, aligned based on the 11 amino acids of the C-terminus (dashed oval).

```
                       1        10        20        30        40        50        60
αo1_human            1 MGCTLSAEERAALERSKAIEKNLKEDGISAAKDVKLLLLGAGESGKSTIVKQMKIIHEDG
αt1_bovine           1 MGAGASAE----EKHSRELEKKLKEDAEKDARTVKLLLLGAGESGKSTIVKQMKIIHQDG
mini-Go              1 -----------------GIEKNLKEDGISAAKDVKLLLLGADNSGKSTIVKQMKIIHGGS
                                         :**:****. . *: ********.:************** ..

                       61       70        80        90        100       110       120
αo1_human           61 FSGEDVKQYKPVVYSNTIQSLAAIVRAMDTLGIEYGDKERKADAKMVCDVVSRMEDTEPF
αt1_bovine          57 YSLEECLEFIAIIYGNTLQSILAIVRAMTTLNIQYGDSARQDDARKLMHMADTIEE-GTM
mini-Go             44 GG----------------------------------------------------------
                        .

                       121      130       140       150       160       170       180
αo1_human          121 SAELLSAMMRLWGDSGIQECFNRSREYQLNDSAKYYLDSLDRIGAADYQPTEQDILRTRV
αt1_bovine         116 PKEMSDIIQRLWKDSGIQACFDRASEYQLNDSAGYYLSDLERLVTPGYVPTEQDVLRSRV
mini-Go             46 ----------------------------------------------------------SG

                       181      190       200       210       220       230       240
αo1_human          181 KTTGIVETHFTFKNLHFRLFDVGGQRSERKKWIHCFEDVTAIIFCVALSGYDQVLHEDET
αt1_bovine         176 KTTGIIETQFSFKDLNFRMFDVGGQRSERKKWIHCFEGVTCIIFIAALSAYDMVLVEDDE
mini-Go             48 GTTGIVETHFTFKNLHFRLFDVGGQRSERKKWIHCFEDVTAIIFCVDLSDY---------
                        ****:**:*:**:*:**:******************.**.*** . ** *

                       241      250       260       270       280       290       300
αo1_human          241 TNRMHESLMLFDSICNNKFFIDTSIILFLNKKDLFGEKIKKSPLTICFPEYTGPNTYEDA
αt1_bovine         236 VNRMHESLHLFNSICNHRYFATTSIVLFLNKKDVFSEKIKKAHLSICFPDYNGPNTYEDA
mini-Go             99 -NRMHESLMDFDSICNNKFFIDTSIILFLNKKDLFGEKIKKSPLTICFPEYTGPNTYEDA
                        *******  *:****:::*  ***:*******:*.*****: *:****:*.********

                       301      310        320       330       340       350 354
αo1_human          301 AAYIQAQFESK-NRSPNKEIYCHMTCATDTNNIQVVFDAVTDIIIANNLRGCGLY
αt1_bovine         296 GNYIKVQFLELNMRRDVKEIYSHMTCATDTQNVKFVFDAVTDIIIKENLKDCGLF
mini-Go            158 AAYIQAQFESK-NRSPNKEIYCHMTCATDTNNAQVIFDAVTDIIIANNLRGCGLY
                       . **:.** .   *   ****.********:* :.:********* :**:.***:
αt_peptide_4A4M                                                    ILENLKDCGLF
```

**Fig. S5. Sequence alignment of $G_{i/o}$ proteins, mini-$G_o$, and $G_t$ peptide.** The alignment is performed using the UniProt server (http://www.uniprot.org/align/).

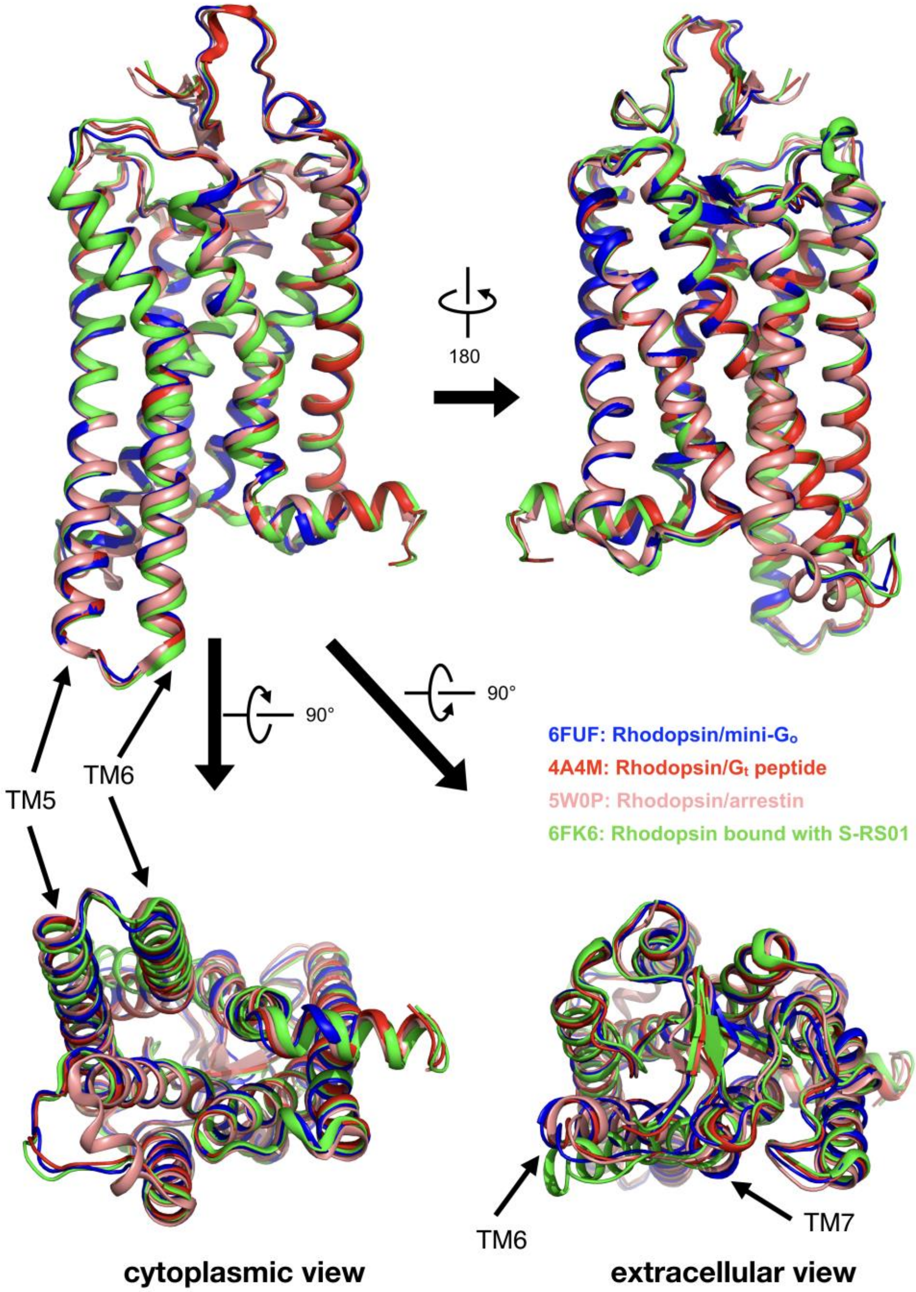


**Fig. S6. Comparison of active rhodopsin structures.** Four active rhodopsin structures are compared: rhodopsin bound to mini-$G_o$ (PDB ID: 6FUF, blue), $G_t$ C-terminal peptide (PDB ID: 4A4M, red), arrestin (PDB ID: 5W0P, salmon pink), and stabilized by the compound S-RS01 (PDB ID: 6FK6, green). Rhodopsin structures are superposed to the Cα atoms of the rhodopsin structure bound with mini-$G_o$.

**Table S1. Statistics of the crystallographic data and the structural refinement.**

| **Data collection** | | |
|---|---|---|
| Space Group | | $p6_1$ |
| Cell dimensions: | a, b, c | 151.36 Å, 151.36 Å, 96.65 Å; |
| | α, β, γ | 90.00°, 90.00°, 120.00° |
| Wavelength (Å) | | 1.0 |
| Resolution* (Å) before STARANISO‡ | | |
| | hk-plane | 3.69 |
| | l-direction | 3.11 |
| | Overall | 49.54 – 3.11 |
| Resolution* (Å) after STARANISO‡ | | 49.544 – 3.020 (3.508 – 3.020) |
| Rpim | | 0.060 (0.930) |
| I/σI | | 9.416 (1.937) |
| Spherical completeness | | 0.5799 (0.0803) |
| Ellipsoidal completeness | | 0.9299 (0.6477) |
| Multiplicity | | 37.31 (30.73) |
| CC(1/2) | | 0.9997 (0.8612) |

| **Refinement** | | |
|---|---|---|
| Refinement program | | PHENIX 1.13_2998 |
| Resolution (Å) | | 49.544 – 3.117 (3.218 – 3.117) |
| No. Reflections | | 14851 |
| $R_{work}$ / $R_{free}$ | | 0.2568 (0.3411) / 0.2801 (0.3516) |
| Number of atoms | | 4008 |
| | Protein | 3973 |
| | All-trans retinal/NAG | 20/14 |
| | Solvent | 1 |
| Ramachandran favoured (%) | | 93.48 |
| Ramachandran allowed (%) | | 6.31 |
| Ramachandran outliers (%) | | 0.20 |
| R.m.s.d. Bond length (Å) | | 0.008 |
| R.m.s.d. Bond angles (°) | | 1.32 |
| Fo, Fc correlation | | 0.86 |
| Anisotropy | | 0.104 |
| Averaged B factor ($Å^2$) | | 93.0 |

Numbers in parentheses are for the highest resolution shell. The glycosylation product, *N*-acetylglucosamine, is abbreviated to NAG.

*) Resolution is defined if CCano is greater than 0.15.

‡) STARANISO server applies anisotropic mask, scales and merges the datasets.

**Table S2. Quantitative measure of similarity between active rhodopsin structures.** The upper value shows the root-mean-square deviation of Cα positions between two different rhodopsin structures. The lower value is the number of Cα atoms used for calculation. The comparison was performed using the *super* command in PyMOL. For rhodopsin/arrestin chimeras, only the rhodopsin part was taken into account. When there are more than one rhodopsin molecules in the PDB file, only the chain A is used for comparison.

| | 6FUF | 2X72 | 4A4M | 4BEY | 4BEZ | 5DYS | 5EN0 | 3CAP | 3DQB | 3PQR | 3PXO | 4J4Q | 4PXF | 4X1H | 5TE3 | 4ZWJ | 5W0P |
|---|---|---|---|---|---|---|---|---|---|---|---|---|---|---|---|---|---|
| **6FUF<br>Rho/mini-Go** | x | 0.482<br>266 | 0.476<br>269 | 0.495<br>268 | 0.531<br>275 | 0.487<br>268 | 0.473<br>268 | 0.564<br>272 | 0.533<br>273 | 0.453<br>268 | 0.484<br>267 | 0.447<br>264 | 0.472<br>266 | 0.407<br>251 | 0.554<br>271 | 0.650<br>288 | 0.631<br>290 |
| **Recombinant bovine rhodopsin, constitutively active mutants, active state** | | | | | | | | | | | | | | | | | |
| **2X72<br>Bovine, N2D/E113Q/D282C<br>All-trans retinal<br>GNAT1 peptide** | *0.482<br>266* | x | 0.267<br>311 | 0.278<br>320 | 0.290<br>315 | 0.365<br>300 | 0.307<br>315 | 0.284<br>272 | 0.477<br>309 | 0.304<br>283 | 0.372<br>284 | 0.282<br>300 | 0.265<br>304 | 0.241<br>295 | 0.271<br>263 | 0.533<br>297 | 0.577<br>291 |
| **4A4M<br>Bovine, N2C/M257Y/D282C<br>All-trans retinal<br>GNAT3 peptide** | *0.476<br>269* | *0.267<br>311* | x | 0.239<br>321 | 0.249<br>315 | 0.325<br>297 | 0.253<br>306 | 0.306<br>281 | 0.350<br>302 | 0.265<br>301 | 0.319<br>289 | 0.255<br>307 | 0.286<br>306 | 0.239<br>301 | 0.310<br>277 | 0.577<br>303 | 0.617<br>299 |
| **4BEY<br>Bovine, N2C/G90D/D282C<br>Retinal isomers<br>GNAT1 peptide** | *0.495<br>268* | *0.278<br>320* | *0.239<br>321* | x | 0.076<br>271 | 0.301<br>290 | 0.246<br>313 | 0.283<br>274 | 0.387<br>308 | 0.247<br>294 | 0.309<br>281 | 0.229<br>310 | 0.266<br>313 | 0.230<br>304 | 0.289<br>281 | 0.562<br>302 | 0.591<br>299 |
| **4BEZ<br>Bovine, N2C/G90D/D282C<br>Retinal isomers<br>No peptide** | *0.531<br>275* | *0.290<br>315* | *0.249<br>315* | *0.076<br>271* | x | 0.298<br>295 | 0.258<br>316 | 0.269<br>278 | 0.407<br>304 | 0.273<br>296 | 0.304<br>286 | 0.254<br>307 | 0.259<br>305 | 0.255<br>302 | 0.249<br>273 | 0.580<br>304 | 0.614<br>301 |
| **5DYS<br>Bovine, N2C/T94I/D282C<br>All-trans retinal<br>No peptide** | *0.487<br>268* | *0.365<br>300* | *0.325<br>297* | *0.301<br>290* | *0.298<br>295* | x | 0.172<br>267 | 0.266<br>289 | 0.444<br>296 | 0.208<br>263 | 0.171<br>295 | 0.260<br>275 | 0.266<br>283 | 0.253<br>272 | 0.203<br>272 | 0.593<br>292 | 0.652<br>295 |
| **5EN0<br>Bovine, N2C/T94I/D282C<br>All-trans retinal<br>GNAT3 peptide** | *0.473<br>268* | *0.307<br>315* | *0.253<br>306* | *0.246<br>313* | *0.258<br>316* | *0.172<br>267* | x | 0.319<br>289 | 0.353<br>294 | 0.159<br>277 | 0.220<br>266 | 0.213<br>311 | 0.232<br>307 | 0.205<br>296 | 0.261<br>270 | 0.516<br>298 | 0.565<br>300 |
| **Native bovine rhodopsin, active state** | | | | | | | | | | | | | | | | | |
| **3CAP<br>Bovine, native<br>Ligand-free<br>No peptide** | *0.564<br>272* | *0.284<br>272* | *0.306<br>281* | *0.283<br>274* | *0.269<br>278* | *0.266<br>289* | *0.319<br>289* | x | 0.484<br>297 | 0.315<br>278 | 0.275<br>281 | 0.273<br>277 | 0.217<br>274 | 0.240<br>263 | 0.181<br>284 | 0.613<br>291 | 0.679<br>293 |
| **3DQB<br>Bovine, native<br>Ligand-free<br>GNAT1 peptide** | *0.533<br>273* | *0.477<br>309* | *0.350<br>302* | *0.387<br>308* | *0.407<br>304* | *0.444<br>296* | *0.353<br>294* | *0.484<br>297* | x | 0.339<br>304 | 0.405<br>297 | 0.317<br>301 | 0.396<br>298 | 0.363<br>301 | 0.496<br>297 | 0.618<br>298 | 0.664<br>303 |
| **3PQR<br>Bovine, native<br>All-trans retinal<br>GNAT1 peptide** | *0.453<br>268* | *0.304<br>283* | *0.265<br>301* | *0.247<br>294* | *0.273<br>296* | *0.208<br>263* | *0.159<br>277* | *0.315<br>278* | *0.339<br>304* | x | 0.157<br>258 | 0.156<br>286 | 0.238<br>290 | 0.202<br>292 | 0.304<br>281 | 0.565<br>299 | 0.583<br>292 |
| **3PXO<br>Bovine, native<br>All-trans retinal<br>No peptide** | *0.484<br>267* | *0.372<br>284* | *0.319<br>289* | *0.309<br>281* | *0.304<br>286* | *0.171<br>295* | *0.220<br>266* | *0.275<br>281* | *0.405<br>297* | *0.157<br>258* | x | 0.250<br>268 | 0.294<br>286 | 0.277<br>275 | 0.252<br>280 | 0.659<br>302 | 0.665<br>295 |
| **4J4Q<br>Bovine, native<br>OG = ligand<br>GNAT1 peptide** | *0.447<br>264* | *0.282<br>300* | *0.255<br>307* | *0.229<br>310* | *0.254<br>307* | *0.260<br>275* | *0.213<br>311* | *0.273<br>277* | *0.317<br>301* | *0.156<br>286* | *0.250<br>268* | x | 0.216<br>300 | 0.150<br>294 | 0.268<br>278 | 0.544<br>298 | 0.568<br>293 |
| **4PXF<br>Bovine, native<br>OG = ligand<br>SAG peptide** | *0.472<br>266* | *0.265<br>304* | *0.286<br>306* | *0.266<br>313* | *0.259<br>305* | *0.266<br>283* | *0.232<br>307* | *0.217<br>274* | *0.396<br>298* | *0.238<br>290* | *0.294<br>286* | *0.216<br>300* | x | 0.180<br>281 | 0.190<br>259 | 0.537<br>301 | 0.559<br>292 |
| **4X1H<br>Bovine, native<br>NG - ligand<br>High-affinity Gt peptide** | *0.407<br>251* | *0.241<br>295* | *0.239<br>301* | *0.230<br>304* | *0.255<br>302* | *0.253<br>272* | *0.205<br>296* | *0.240<br>263* | *0.363<br>301* | *0.202<br>292* | *0.277<br>275* | *0.150<br>294* | *0.180<br>281* | x | 0.216<br>253 | 0.528<br>291 | 0.573<br>293 |
| **5TE3<br>Bovine, native<br>OG = ligand<br>No peptide** | *0.554<br>271* | *0.271<br>263* | *0.310<br>277* | *0.289<br>281* | *0.249<br>273* | *0.203<br>272* | *0.261<br>270* | *0.181<br>284* | *0.496<br>297* | *0.304<br>281* | *0.252<br>280* | *0.268<br>278* | *0.190<br>259* | *0.216<br>253* | x | 0.592<br>288 | 0.684<br>278 |
| **Human rhodopsin, T4-lysozyme/Arrestin chimera, active state** | | | | | | | | | | | | | | | | | |
| **4ZWJ<br>Human, N2C/E113Q/M257Y/D282C<br>Ligand-free<br>T4/Arrestin chimera** | *0.650<br>288* | *0.533<br>297* | *0.577<br>303* | *0.562<br>302* | *0.580<br>304* | *0.593<br>292* | *0.516<br>298* | *0.613<br>291* | *0.618<br>298* | *0.565<br>299* | *0.659<br>302* | *0.544<br>298* | *0.537<br>301* | *0.528<br>291* | *0.592<br>288* | x | 0.342<br>299 |
| **5W0P<br>Human, N2C/E113Q/M257Y/D282C<br>Ligand-free<br>T4/Arrestin chimera** | *0.631<br>290* | *0.577<br>291* | *0.617<br>299* | *0.591<br>299* | *0.614<br>301* | *0.652<br>295* | *0.565<br>300* | *0.679<br>293* | *0.664<br>303* | *0.583<br>292* | *0.665<br>295* | *0.568<br>293* | *0.559<br>292* | *0.573<br>293* | *0.684<br>278* | *0.342<br>299* | x |
| **Recombinant bovine rhodopsin, thermalstable mutant, inactive state** | | | | | | | | | | | | | | | | | |
| **2J4Y<br>Bovine, N2C/D282C<br>11-cis retinal<br>No peptide** | 1.546<br>278 | 1.612<br>293 | 1.655<br>294 | 1.636<br>295 | 1.613<br>296 | 1.498<br>288 | 1.545<br>290 | 1.533<br>294 | 1.634<br>292 | 1.560<br>288 | 1.538<br>290 | 1.585<br>293 | 1.575<br>294 | 1.578<br>288 | 1.455<br>291 | 1.491<br>281 | 1.470<br>280 |
